# A Bibliometric Analysis on Conservation Land Trust and Implication for China

**DOI:** 10.3390/ijerph191912741

**Published:** 2022-10-05

**Authors:** Chuan Yang, Mingfeng Li, Ziqi Wang

**Affiliations:** School of Public Administration, Central China Normal University, Wuhan 430079, China

**Keywords:** land trust, conservation easement, land conservation, bibliometrics, VOSviewer, CiteSpace, biodiversity

## Abstract

Modern land protection and biodiversity conservation depend greatly on the application of land trust. With the accelerated development of land trust organizations, the land trust has become the most effective land conservation method. Land trusts have been widely used in the ecological protection of nature reserves abroad and have achieved remarkable results. The research on international land trusts has important reference value for the innovation of ecological protection models of China’s nature reserves. This study aims to explore the research hotspots of international land trust conservation, clarify the evolution of related knowledge, and provide a reference for domestically related theoretical research and practical work. The research results show the following: (1) From the perspective of the publication trend, the number of international research publications increased exponentially from 1997 to 2021, and the research involves a wide range of disciplines. (2) In terms of research hotspots, land trust and private land protection, the impact of and response to climate change, and the trust mechanism in collaborative governance constitute three hot research topics. (3) From the knowledge base of the research, the international land trust research has formed seven knowledge clusters with clear boundaries, and five key documents with the theme of conservation easements constitute an important knowledge base in this field. (4) Land trust conservation easement as private law can effectively make up for the deficiency of public law. These findings can provide help and reference for domestic land conservation, as well as the reform of China’s ecological civilization system.

## 1. Introduction

Land is an important space carrier for ecological civilization construction, the most basic production element of socio-economic development, and the most fundamental habitat for wildlife and rare species. In the past few decades, with the development of urbanization, a large number of people poured into cities, which directly led to the reduction in human natural leisure activities, resulting in the spatial isolation of people and the natural environment [1]. This isolation will have a series of serious consequences, including a deterioration in public health and well-being, a decline in natural affinity, and a lack of pro-environmental behaviors among the population [2]. Re-establishing the connection between humans and nature is crucial to achieving healthy social development and addressing widespread environmental problems. Establishing extensive protected areas is an important tool for people in responding to the biodiversity crisis. In countries such as the United States, land trust is an essential part of biodiversity conservation and a viable or complementary mechanism to public administration for biodiversity conservation. Despite the diversity of incentives for and approaches to conserving private land, land trusts stand out as the best-organized and fastest-growing [3]. 

Legally, a trust is an arrangement in which one party holds property for the benefit of another. A land trust is just one of various trusts. Various land trusts have diverse interests. Today’s land trust is widely used in conserving the ecological values of land; protecting historical, agricultural, and recreational land; and raising money for real estate development. Specifically, our research focuses on non-profit or charitable organizations that protect nature preserves through land trusts. Natural resources protected and managed by such organizations include forests, wetlands, farms, and natural habitats, as well as areas that hold water sources, cultural landmarks, or even landscapes. In 1891, Charles founded a personal voluntary organization called “Trustees of Reservations” in Massachusetts to protect land with important ecological functions, beautiful landscapes, and historical significance by purchasing land and then opening it to the public. This was the first land trust project in the world. Today, land trusts have become the most effective form of land protection in the United States and an integral part of the environmental protection system [4]. At the end of 2020, over 61 million acres of land have been protected by the land trust, and approximately 70% of the newly protected land from 2015 to 2020 benefited from the land trust [5]. Through years of development, the United States has become the most mature country in the land trust area. In developed countries such as Australia, Canada, and England, land trusts have also played an important role in the ecological protection of nature reserves [6,7,8].

Conservation easement agreements frequently provide landowners with financial compensation or tax breaks in exchange for the social and public benefits provided by the land (e.g., open space, ecological services). It is undeniable that land trusts use economic means to protect the inherent advantages of land. A land trust can facilitate interaction between different stakeholders and coordinate community residents, local governments, and landowners cooperating in conservation actions and achieving conservation goals, such as working with landowners to reduce the social and economic costs of land use [9]. Some scholars, however, have also questioned the preferential tax policies for conservation easements. Rather than incentivizing the landowner’s conservation behavior, the tax breaks may only benefit the wealthy landowner [10]. In addition, the land trust has been the fastest-growing and most effective mode of protection in the past few decades. Protecting private land by conservation easements has become an effective alternative to underfunded and controversial public acquisitions [3,11]. 

Nowadays, the Chinese government protects natural resources mainly by means of public law, which cannot mobilize the initiative of land users. Meanwhile, China’s current land protection system is an administrative system led by the government at multiple levels of planning and supervision. It not only concentrates powers and responsibilities on the government, but also relies too much on mandatory land use control, resulting in high management costs and low operational efficiency [12]. In response to the problems, the State Council of China proposed to “learn from international experience” and “establish and improve a long-term mechanism for the government, enterprises, social organizations and the public to participate in nature conservation” [13]. In the practice of land trust conservation in developed countries, the government, enterprises, and social organizations participate together, and financial institutions provide financing support, which has important reference significance for the innovation of ecological environmental protection models in China’s natural reserves. While the land trust is rapidly evolving, the topic of land trusts has been a source of concern among scholars, and a great number of study findings have been published. This article aims to better grasp the development trend of land trust issues and provides a useful reference for the theoretical research and practical work of China’s nature reserve land trusts. A comprehensive scientometric analysis of land trust literature was provided to (1) analyze the publishing trend of literature in the field of land trust and visualize the research performance of countries; (2) use the bibliometrics method to present the hot topics in land trust research; (3) from the references, identify the core literature and visualize the trends and frontiers; and (4) draw implications for China’s scenario. Overall, this paper provides the panorama of land trust research for scholars and policymakers.

## 2. Materials and Methods

### 2.1. Scientometric Analysis Method

This study uses VOSviewer (Centre for Science and Technology Studies, Leiden University, The Netherlands) and CiteSpace software (Drexel University, Philadelphia, PA, USA) to map the knowledge of the land trust research literature over the last 30 years. The CiteSpace software, based on the Java language, was first developed by Dr. Chaomei Chen from the School of Information Science and Technology of Drexel University and the WISE Laboratory of Dalian University of Technology [14]. This software can show the development trends of a certain knowledge field in a visual knowledge map by extracting and calculating the literature information. It offers eight different visualization graphs to illustrate different development trends of a discipline. It has widely been used to identify research frontiers and knowledge bases and has become popular in scientometric research. The VOSviewer software developed by Leiden University in the Netherlands can dig up the underlying information through bibliometrics and can also present visual analysis by developing citation networks and co-occurrence networks [15]. When conducting bibliometrics analysis, the VOSviewer can clearly show the relationship between subject topics, and CiteSpace has more advantages in revealing the discipline development trends. We use VOSviewer and CiteSpace extensively to generate a visual interpretation of land trust literature from the last 30 years due to their robust data processing and visualization functions. This paper uses the methods of keyword co-occurrence and reference co-citation analysis to study the intellectual structures of land trust research. Both keyword co-occurrence and reference co-citation are based on the calculation of co-occurrence analysis, which was used to measure the frequency of important keywords and valuable articles. The purpose of co-occurrence analysis is to explore changes in research hotspots in such disciplines, and the reference co-citation tends to be employed to find the knowledge bases.

### 2.2. Data Collection

**Figure 1:**
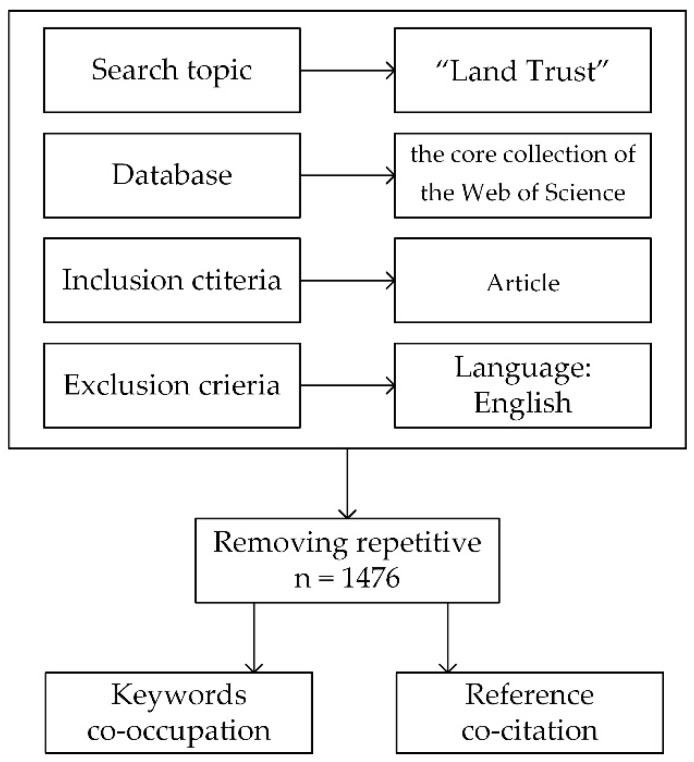
Flow chart for the research process.

To ensure the reliability of the literature data, we selected the core collection of the Web of Science as the literature source. The Web of Science is an important academic database, for it contains representative and cutting-edge literature covering fields such as social science, natural science, art, and the humanities. More importantly, the Web of Science database is perfectly compatible with VOSviewer and CiteSpace. In the process of retrieval, we took “Land Trust” as the search topic. The other criteria were set as follows: language = English, document type = article, and time span from 1990 to 2021. To ensure the accuracy and authenticity of the obtained literature, we read the titles and the abstracts of the collected papers to ensure these papers were relevant to the research topic. Finally, as shown in Figure 1, a total of 1476 results were obtained, each of which included the title, author, keywords, references, abstract, DOI, and other information. From the perspective of Web of Science category distribution, the collected literature is mainly distributed in environmental science, ecology, planning, geography, economics, management, law, and other disciplines, and most of the research is related to the two disciplines of environmental science and ecology.

## 3. Results

### 3.1. An Overview of Land Trust Research

**Figure 2:**
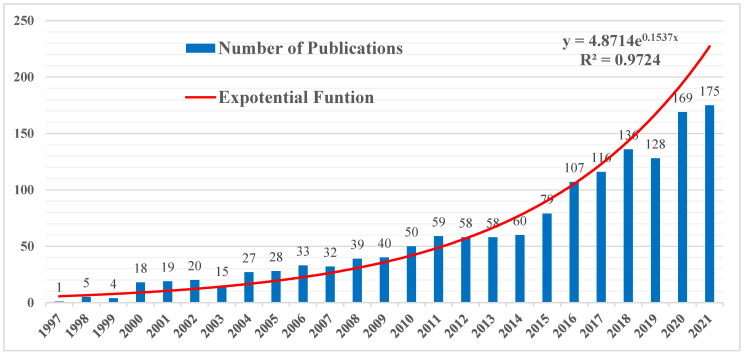
The annual number of publications related to the land trust field.

The year-by-year distribution of published literature concerning land trusts from 1990 to 2021 is shown in Figure 2. During the search period, the number of publications related to land trusts generally increased exponentially. The blue bar shows the number of annual publications, and the red line illustrates the growth trend that is fitted with an exponential growth model. Based on the method of natural breaks, we can divide the research distribution into three stages, as follows:

Low-speed fluctuating increase stage (1997–2007): The retrieval time span was 1990 to 2021, but there were no articles found before in the years from 1990 to 1996, so they are not shown in the graph. It can be seen from Figure 2 that the first paper was published in 1997, after which the number of published articles gradually increased to 32 in 2007. Overall, the increase in the number of research papers is mainly due to the explosive growth of land trust development. In the 1980s, the land trust became a popular land protection mode, and the use of conservation easement has increased dramatically. The area of newly added conservation land trust in the United States rose from less than 160,000 acres in 1995 to more than 800,000 acres in 2003 [16]. The land trust research at this stage mainly focuses on the discussion of the application scenarios of land trusts [17,18].

Accelerated growth stage (2008–2015): This time period is the stage of rapid development of land trust research. With the continuous expansion of the protected area of land trusts in the United States, land trusts have increasingly become the focus of public attention and a hotspot of academic research. The number of relevant research papers has grown rapidly, and the number of publications has increased from 39 in 2008 to 79 in 2015. The research at this stage focused on the construction and improvement of the land trust system [19,20].

Stage of stable and rapid growth (2016–2021): In 2016, the number of publications exceeded 100. Although the number of papers dropped slightly in 2019, the trend line showed that the publications tended to be stable and maintained at a high level. The final stage is the maturity stage of the research field. The focus of land trust research has gradually shifted to the micro-analysis of trust cases. Scholars have used a variety of research methods to conduct a large number of empirical studies on conservation land trust cases in various countries from multiple disciplines and perspectives [21,22], showing the interdisciplinary characteristics of land trust research.

**Figure 3:**
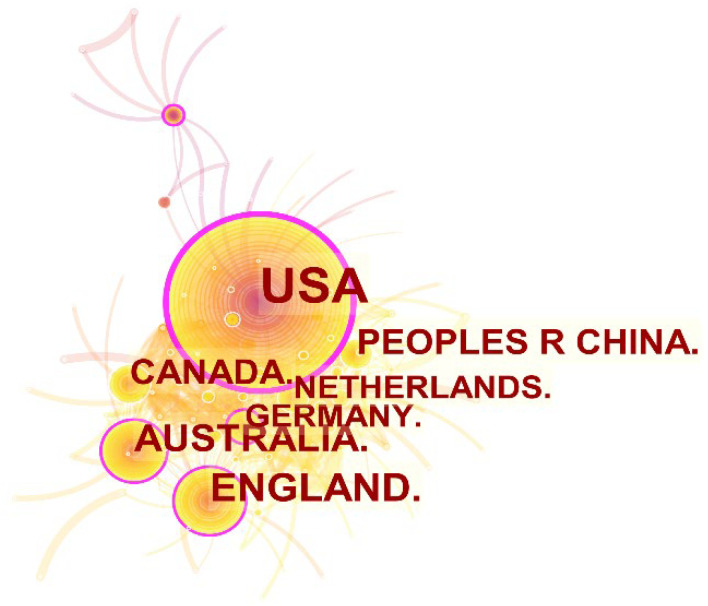
A visualization of the countries’ collaboration network.

In order to obtain more information about countries, the graph of the global distribution of land trust research was generated by CiteSpace. As shown in Figure 3, the research network includes 145 nodes and 601 links. Each node represents a country, and the links indicate the relationships between them. The size of each node is proportional to the publication volume, while the connecting lines between them demonstrate their cooperation. The “countries” refer to the research institutions or affiliations of the authors. Furthermore, the purple outer circle of the node means a key country with a betweenness centrality above 0.1 [23]. Among them, the USA ranked first by publishing 590 articles, followed by England and Australia. It seems that these countries form a leading land trust research group. As is well known, trusts originated in England and gradually gained maturity in the USA, attracting a large number of scholars to investigate land trusts in theories and practice. Therefore, land trust research was dominated by developed countries. The top four countries in terms of centrality above 0.1 were the USA, England, Australia, and Germany. Conservation land trusts originated in the United States, as was previously noted, and were afterward implemented in other industrialized nations, drawing many academics and professionals to conduct research in this field. It is noteworthy that China is the only developing nation in Figure 3, but the absence of line connections with other nations may be due to China still being in the early stages of land trust protection. As a result, there is not much research collaboration between Chinese and foreign scholars in the area of conservation easement. 

### 3.2. Thematic Focus Areas

**Figure 4:**
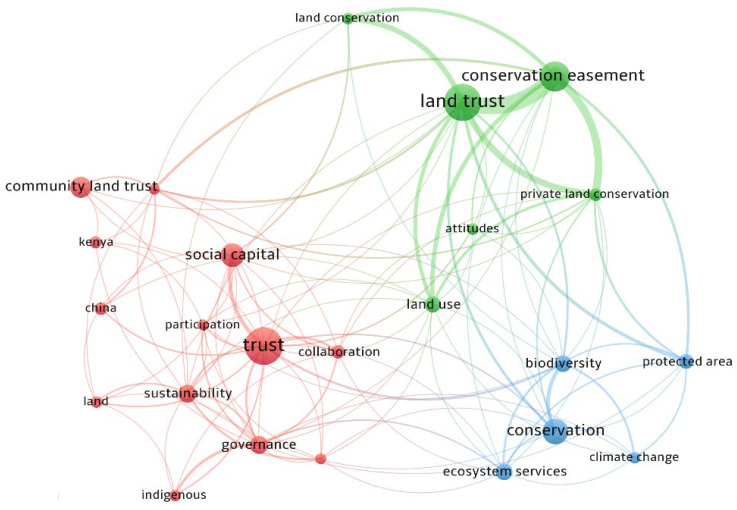
The keywords’ co-occurrence in the land trust field.

The author of an article will typically employ keywords to characterize the research’s essential topic. In general, if a keyword appears frequently throughout a certain period, it means that the keyword reflects a hot issue in the study area at the time. The VOSviewer software’s keyword co-occurrence analysis can be used to extract keyword frequencies as well as detect and present co-occurrence associations in a graphical format. We merged the synonyms and kept the high-frequency keywords to make the map more readable. By introducing the collected 1476 records into VOSviewer, the keyword co-occurrence map shown in Figure 4 was obtained, and the keyword co-occurrence calculation shown in Table 1 was obtained. It can be observed that three major clusters can be identified that are shown using three different colors. The research hotspots of conservation land trusts in nature reserves mainly focus on three aspects: land trust and private land protection (the green part in Figure 4), climate change impact and response (the blue part in Figure 4), and trust mechanism in collaborative governance (the red part in Figure 4). 

This green cluster’s main research area is the protection of private land through conservation easements. The high-frequency keywords in the green cluster are land trust, conservation easement, land use, private land conservation, land conservation, and attitudes. Among them, “conservation easement” is the most closely related to the search term “land trust”. The relevant literature discusses the importance of private land eco-protection from different perspectives and examines the relevant practical application of conservation easements in private land protection. The changing of the world’s landscape is increasing rapidly, which has led to the extinction of rare species and the loss of natural reserves [24]. Most of the established nature reserves in the United States were located in high-altitude areas with poor soil, while more than 90% of endangered species live on private land with fertile soil at low altitudes, which indicated that ecosystem protection on private land should be strengthened [25]. Due to the limited budget for government land protection, traditional protection methods such as government acquisition and land use regulation have been unable to meet the needs of land protection [26]. Non-government organizations respond promptly to this situation [27]. Land trusts use conservation easement and other tools to protect private land, landscapes, nature reserves, and community open space. The cost of land acquisition and land use transformation has a huge impact on land conservation benefits. So, it is important to improve the reserve location strategy to minimize the biodiversity loss caused by future land use change [28]. The “attitude” refers to people’s understanding of land trusts at different levels. It is generally assumed that attitudes will drive individuals to participate in land trust conservation. Considering the lack of public awareness of land trusts and the low enthusiasm for participation, it is suggested that land trust organizations enhance public understanding and support of land trusts by increasing publicity and transparency of the use of land trust property [29]. 

This blue cluster’s main research area is the impact of and response to climate change. The high-frequency keywords in the blue cluster include conservation, biodiversity, ecosystem services, protected area, and climate change. There are relatively few keywords in this cluster on the map (Figure 4), but there are many links between the keywords such as protected areas and biodiversity and the keywords in the green cluster, which demonstrates a relatively close relationship between these two clusters. The related articles in the blue cluster mainly discuss the impact of global climate change on the ecosystems of protected areas. The local governments in the United States, along with land trusts and other non-governmental organizations (NGOs), have raised a large amount of financial resources to protect nature reserves. On the one hand, these conserved reserves can buffer communities from the impact of climate change, thereby improving resilience [30]. On the other hand, climate impacts also threaten nature conservation. Reserves will be affected by storms, extreme temperatures, and other factors [31]. Natural land in coastal areas provides communities with various ecosystem services, such as temperature regulation, carbon storage, and water conservation. However, as the sea level continues to rise at an average of 3 mm per year, it is predicted that by 2100, about a quarter of the natural reserves in the eastern United States will be affected by sea level rise. As a result, Epanchin proposed near-term coast protection from seawater erosion by purifying runoff, as well as long-term ecological corridors to assist affected organisms in moving inward land transfer [32]. Moreover, lacking plans and lacking funding were the greatest challenges in some states. Although permanent easements can protect the ecological environment by restricting land development, the permanence clause also limits the right of land managers to flexibly adjust conservation plans in response to climate change. Therefore, the temporal and spatial perpetual protection clauses in the traditional easement agreement should be adjusted to the term of protection, and the spatial location of protection can be flexibly adjusted according to climate change [33,34].

This red cluster’s main research area is the cooperative trust mechanism in land protection. The main keywords of the cluster include trust, social capital, community land trust, sustainability, governance, collaboration, China, Kenya, land, indigenous, agriculture, property rights, and participation. Land protection means the supervision and restriction of land use. With the continuous expansion of the scale of land trust protection, the central governments of some Western developed countries have gradually delegated the responsibility of natural resource protection to local governments and NGOs. As an alternative to traditional preservation strategies, a land trust can be viewed as a tool for landowners and easement holders in collaborating to safeguard ecological resources, and the social relationship between them has a direct impact on the land trust’s implementation effect. Scholars have focused on the trust relationship between land trust settlor and trustee and its impact on the protection effect of land trusts in this context.

In terms of theoretical research, Stern and Coleman used normative research methods to analyze the antecedents of four dimensions of trust, namely dispositional trust, rational trust, affinity trust, and procedural trust, and discussed the potential impact of different dimensions of trust on natural resource management. They pointed out that if natural resource protection agencies can gain public trust in one or several dimensions, they can effectively reduce the resistance to natural resource protection work [35]. In terms of empirical research, Mase et al. found that among different types of nature conservation institutions, the public’s trust in land trust institutions is lower than that in university branches and government departments. The reason is that the public is relatively unfamiliar with the model of protecting natural ecology through a land trust. Then, they proposed that land trust institutions should strengthen communication and interaction with the public to enhance the public’s understanding of land trust [36]. Social capital can also be used to promote conservation efforts. For example, social networks can aid in the coordination of natural resource management efforts and assist farmers in better adapting to climate change policies, and the formation of a trust mechanism improves the efficiency of conservation initiatives [37].

### 3.3. Document Co-Citation Analysis

When two or more documents appear in the third bibliographical reference and go on to form a co-citation relationship, this is referred to as co-citation. This relationship can be used to examine the growth and development of a discipline. In a certain research field, a series of highly cited articles form the knowledge base of research [38]. In literature research, analyzing the key clusters and nodes in the literature co-citation network can help reveal the important information contained in the key literature in the evolution of research in a field [39]. We conducted reference co-citation analysis to reveal the intellectual structures of the land trust domain and disclose its knowledge roots. In the process, knowledge base clusters were also identified, which could help reflect the trends in the scientific evolution of this field. The CiteSpace parameters were used as follows: We select the highest number of times per year cited 50 articles in CiteSpace to build the co-citation network map, and then merge the maps of each year. In order to prevent the atlas from being too confused, the pathfinder algorithm was used to cluster the maps. Finally, the LSI algorithm was used to extract keywords from clusters, and then main clusters containing more than 30 documents were retained.

Seven primary clusters can be shown as the most represented research direction in the area of land trust when combined with the representative title, abstract, and keyword in a preliminary analysis. Table 2 displays specific details about clusters. The silhouette value, also known as homogeneity, reveals how similar the documents in the clusters are, while the cluster size tells how many documents are present in the cluster. The degree of consistency of document subjects in clustering increases as the silhouette value becomes closer to 1.

**Figure 5:**
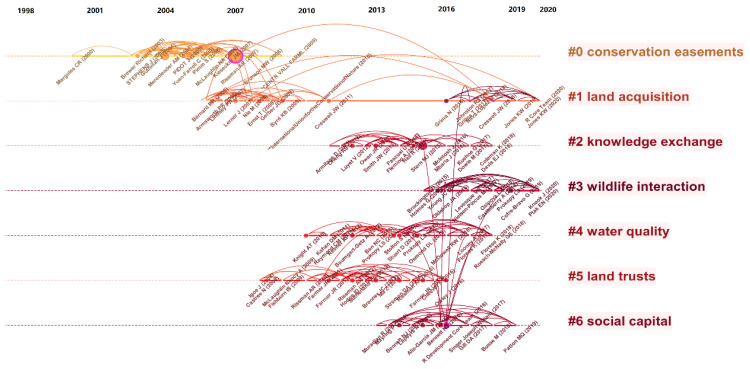
Timeline clustering map of the articles’ co-citation network.

In order to reflect the changes in the academic attention of each clustering research article in each period, this study uses the timeline pattern to arrange the clusters from top to bottom according to the size of the clusters. Figure 5 displays the co-citation analysis of 1476 linked land trust research articles published between 1990 and 2021. Each cited document is represented by a node in the clustering map. The co-citation frequency is inversely correlated with node size, and the co-citation link strength is indicated by the link thickness map. The literature that was cited in the literature dataset reflects the cross-relationship between the literature that was cited and the related literature that was cited, which forms the research frontier. Therefore, subject clustering of cited documents can expose the knowledge base and identify the frontier basic knowledge of a co-citation cluster based on the co-citation relationship of the highly cited publications that make up the knowledge base.

According to Figure 5, CiteSpace’s subject clustering map for the land trust study field from 1990 to 2021 shows seven clusters. The research hotspots and the development of land trust issues during the past 30 years are reflected in these clusters. Understanding the study questions for each cluster, establishing the connections between the clusters, and organizing the progress of land trust research are all aided by the extraction and analysis of the literature information in each cluster.

Cluster #0: The research theme of this cluster is conservation easement. Judging from the timeline distribution of cluster #0 in the timeline diagram (Figure 5), we can see that the related studies on conservation easements constitute the beginning of the land trust field. The conservation easement agreement creates a legally binding agreement on the development and utilization of nature reserves, which can limit the future development of land without changing land ownership [40]. Most of the conservation easements in the United States are promoted by local land trusts, which obtain easements through negotiation, donation, and direct purchase while allowing the landowner to retain the property rights and management rights of the land. However, the land owner’s construction, mining, logging, and other destructive land use behaviors are bound by the easement agreement to protect the natural, historical, and economic value [41,42]. The advantage of protecting the land ecology by signing an easement agreement is that the innovation of the land use contract allows the rights to the land on the land conservation to be subdivided so that the land trust agency does not need to pay a high price to purchase land ownership but only needs to obtain a conservation easement at a lower cost to realize the effective protection of the nature reserves [43]. 

In the co-citation analysis of documents, the literature with a high citation burst value deserves attention. Documents with high burst values refer to publications that have a sudden increase in the number of citations in a short period of time. The higher the burst value is, the higher the degree of attention in the corresponding period is, and the stronger the frontier representativeness is. Using the burst detection of CiteSpace, five documents were identified from cluster #0 (Table 3). These five documents retrospect and summarize the development process of land trusts and point out the direction for the improvement and development of the relevant systems for the protection of conservation easements. Fairfax argued that an overemphasis on private land trusts will undermine government efforts to protect the environment and may not be conducive to achieving land conservation goals by reviewing cases of land conservation in the United States over the past 200 years [44]. Pidot also pointed out that the conservation easement is an effective supplement to land conservation policies such as land planning and taxation, but the conservation easement still faced problems such as unclear legal concepts, difficulty in guaranteeing public interests, and difficulty in evaluating protection effects. Therefore, it is necessary to revise the law and improve the conservation easement scheme [45]. Nowadays, most easements are aimed at reducing development to protect the core habitat and biodiversity. Meanwhile, conservation easements also allowed the landowner to preserve a wide range of land for private uses, but there were no clear restrictions on the use of private land. Many studies have pointed out that it is necessary to subdivide the construction projects in the area where the conservation easement is obtained and clarify which types of project construction should be limited [46]. At the same time, it is also important to examine the long-term effect of conservation easements and investigate which type of institution easement work is more effective in a specific ecological and political environment [42]. Moreover, by studying the relationship between the spatial distribution of conservation easements and the effect of easement implementation, a scientific and effective basis for optimizing the spatial distribution of conservation easements can be provided [47].

Cluster #1: The research theme of this cluster is land acquisition. The articles in this cluster were mainly published in two time periods: 2005 to 2010 and 2015 to 2020. Among them, the literature published from 2005 to 2010 mainly focused on how ecological organizations effectively acquire land. According to preliminary estimates, it will cost a total of USD 488.4 billion to establish a comprehensive ecological protection network in the continental United States through direct acquisition of land and USD 256.8 billion through conservation easements [48]. Protecting land through conservation easements has obvious cost advantages. Land acquisition through conservation easement effectively makes up for the shortage of public financial funds. In addition to the direct acquisition of land, the use of land use planning to restrain the behavior of land users can also indirectly achieve the purpose of land protection. According to relevant research, when purchasing protected land, private land trust institutions should strengthen the coordination and communication of local governments on land use planning so as to effectively make up for the lack of public financial funds in land protection and maximize the protection benefits of trust funds [49,50]. 

The literature published between 2015 and 2020 mainly studied the economic cost calculation, ecological performance evaluation, and decision feedback of stakeholders of the payment for ecosystem service (PES) through case studies [51,52]. PES can encourage landowners to protect natural resources while also increasing land cover management activities [53]. A quasi-experiment measured the ecological benefits of the PES project in Veracruz, Mexico, and found that the PES project not only helped reduce deforestation but also achieved benefits in water conservation and carbon storage that greatly exceeded the cost of the PES project [54]. Another group of scholars pointed out that over-reliance on PES as a win–win solution might lead to ineffective outcomes [55]. Economic activities that expect to pay compensation to offset high environmental hazards may cause such a situation. Due to the increased opportunity cost of protection, only increasing the level of compensation can protect the ecosystem. Therefore, there is a requirement to shift the strategy towards a more targeted policy framework, in which PES constitutes only one of the potential solutions, which may be a more effective way to tackle social and environmental challenges.

Cluster #2: The research theme of this cluster is knowledge exchange. This cluster mainly studies the relationship between knowledge acquisition and natural resource conservation. Social learning is an important concept in the field of natural resource management, which emphasizes the impact of learning and imitating the behavior of others on one’s own decision-making. The positive influence of social networks and the demonstration effect of peers around them are conducive to eliminating the doubts of landowners about the effectiveness of ecological protection measures and can lead more people to participate in ecological conservation [37]. Behavior learning among farmers has social characteristics, but this kind of learning may not necessarily improve the governance of the natural environment [56]. As practitioners of land protection actions, land conservation practitioners need to have professional knowledge and mastery of skills. However, most employees in the United States are only familiar with the two protection methods of protection easement and direct compensation plan, and their understanding of different types of land protection organizations is also different. Therefore, it is proposed that targeted training should be strengthened to improve the professional ability of employees to meet the challenges of private land protection [57].

Cluster #3: The research theme of this cluster is wildlife interaction. Although the name of the cluster is not directly related to the land trust in the literal sense, the cluster focuses on the interaction between humans and wildlife in nature reserves and is closely related to the original intention of the land trust to protect biodiversity. There are conflicts of interest between humans and wild animals in nature reserves. Wildlife activities will threaten human life, the economy, and safety. Humans’ growing demand for land resources will seriously squeeze the living space of other species [58]. By 2050, the global farmland area is expected to increase by 200–300 million hectares compared with 2005, and the conflict between humans and wild animals will further intensify [59]. Determining how to improve and alleviate this conflict and reduce the negative impact of human activities is crucial for the protection of biodiversity, the healthy operation of ecosystems, and the sustainable development of human beings. Maintaining existing nature reserves and building new protected areas are key tools to protect biological habitats. In addition to important protection functions, the establishment of protected areas also limits the rights of landowners to use land for profit. Imposing land use restrictions on local people can easily cause dissatisfaction and lead to management failure [60]. The interaction between humans and wildlife is a collective action involving public interests, so wildlife protection agencies need to strengthen their ties with landowners and the public to achieve the goal of protecting wildlife while protecting human safety [61]. 

Cluster #4: The research theme of this cluster is water quality. Land trust conservation has multiple goals, such as biodiversity protection and soil and water conservation. [62]. Whether the conservation action can be carried out smoothly largely depends on whether the landowner agrees with the concept of nature conservation and is willing to transfer all or part of the land rights to the trust agency. Therefore, it is of great significance to study the motivation of the landowner to participate in conservation action [63,64,65]. The literature under this cluster uses a variety of research methods to explore the motivation of farmers to participate in ecological protection plans from a micro perspective. Ranjan compiled and coded relevant documents on farmers’ willingness to participate in soil and water conservation plans from 1982 to 2018 to present a systematic review of all qualitative investigations, and concluded that farmers’ economic and management needs and their perceived and actual limitations to conservation behavior would affect their actions [66]. Baumgart conducted a meta-analysis of the relevant research literature on agricultural best management practices (BMPs) in the United States and found that farmers’ environmental protection awareness and attitude towards environmental protection have an important impact on environmental protection practices [67]. The study also provides methodological guidance on how to collect data scientifically and effectively to advance land conservation practices and guide scientific research. 

Cluster #5: The research theme of this cluster is land trusts. The cluster name is the same as the literature search term. It mainly focuses on the motivation of private participation in land trusts. On the basis of the former cluster, it further studies the impact of the interaction between landowners and NGOs on land conservation. The motivations of the land owner (easement supplier) and the land trust organization (easement demander) in participating in the land trust are similar. The difference between the two motivations may lead to the failure of the negotiation of the protection agreement. The opportunity for both parties to reach an agreement can be improved by reducing transaction costs and improving the acceptance of easements [68]. If the underlying social relationship between landowners and easement holders becomes adversarial rather than cooperative, there is a risk of undermining the value and effectiveness of conservation easements as a legitimate tool to protect private land. Therefore, easement holding institutions should improve the professional quality of practitioners to better provide landowners with services such as information consultation and technical assistance [69]. 

Cluster #6: The research theme of this cluster is social capital. This cluster focuses on the role of social capital in the land management process. Although the government’s promotion of the establishment of protected areas rather than the involvement of social capital may bring positive effects such as mitigating climate and environmental threats and increasing the income of regional leisure tourism, it may also have negative effects such as affecting the livelihood of fishermen, rent-seeking by managers, and boycott by local politicians [70]. This is not conducive to the sustainability of conservation strategies, because successful conservation strategy implementation is influenced not only by local support for conservation actions, but also by local community perceptions of management and governance opinions. One is that individual members’ different single capabilities can be combined to form a stronger collective capability; the other is that a strong social network can provide individuals with more resource support and ability development, as well as promote more in-depth cooperation among people [71]. At the same time, there are also some shortcomings in social cooperation. The involvement of social capital in ecological protection has problems such as low operational efficiency and a lack of benefit-sharing mechanisms. Therefore, it is necessary for relevant institutions to improve the knowledge network at the community level through capacity building and knowledge sharing. At the same time, farmers should be encouraged to increase their participation in land management through collective action [72].

## 4. Discussion

### 4.1. Trends for Land Trust Research

From the timeline distribution of co-cited document clusters (Figure 5), according to the different starting points of attention of each cluster, the research on land trust protection can be divided into three stages: starting stage, development stage, and maturity stage. The starting stage began in 2000 when Margules conducted a study on the systematic conservation planning of nature reserves [73]. After that, the research literature under the theme of conservation easement continued to increase. By 2005, the related research literature showed an explosive growth trend. During this period, a number of key documents with high emergent value emerged (Table 3), which laid the foundation for the research on nature conservation land trusts and effectively led the development of land trust research. From 2006 to 2008, the research on land trust protection began to extend to two research branches, cluster #1 and cluster #5. It is worth noting that cluster #1 has been of interest to the academic community until now, which shows that the research of this cluster has a high guiding value and a leading role in land trust protection research. The maturity stage began around 2010, and the number of clusters that received attention from the academic community at the same time rose to four, indicating that the breadth and depth of research on land trust protection during this period were greatly expanded, and the degree of attention of related literature gradually peaked. After 2018, cluster #1, cluster #3, and cluster #6 were still closely related to the hot and difficult issues in the practice of land trust protection in various countries and still attracted the attention of the academic community. It is foreseeable that the research interest in these clusters will continue.

In general, following years of development, research on the protection of international land trusts has become a multidisciplinary and rich-content knowledge structure. Overall, the conservation easement topic is the oldest research co-citation cluster topic and the main source of the research object of this study. The conservation easement is a market-based instrument that offers financial incentives to landowners in exchange for protecting ecological services on their properties. It is beneficial for academics to re-evaluate the efficiency of conservation easements and regional trends in conservation funds in the course of reaching the aim. On the other hand, it is worthwhile to talk about the various conservation preferences held by landowners and land trust experts, including transaction costs, frequency, and easement acceptance.

### 4.2. Applicability of Land Trust in China

Faced with the increasingly serious degradation of ecological resources and environmental crisis, the Chinese government has incorporated the problem of natural protection into the field of public law management. For example, in the Grassland law, it is explicitly stipulated that reclamation of grasslands is prohibited, and grazing prohibition and grazing rest systems are implemented in ecologically fragile areas. Similar mandatory prohibitions also appear in many other laws and regulations on natural resource management. It is true that the use of public law means to achieve the goal of land protection has the natural advantages of simplicity and implementation through state coercion, but there are also many drawbacks. On the one hand, it lacks the incentive function of private law. The obligee is unable to obtain direct economic benefits from the legally mandated obligations and therefore lacks the enthusiasm to implement the pro-environmental behavior. On the other hand, it does not fully respect the property rights of land users. For instance, the project of returning grazing land to grassland, for instance, is not based on the willingness of herdsmen. Most of them are prohibited from grazing in the corresponding areas after the government has determined the scope of returning grazing land and compensation standards without full democratic consultation with the actual obligee. When the government’s environmental protection goals conflict with the interests of land users, land users often choose to not implement or to respond passively to the government’s land protection orders, which makes it difficult to achieve the government’s ecological protection goals.

The governments of some countries, including China, have been using public laws to force people to protect land, but the land protection effect is not satisfactory. When exploring a balance between natural resource protection and protection of the interests of natural resource rights holders, the easement protection system implemented by the United States and other countries has achieved remarkable results in practice, providing a land protection model for China and other countries [74,75]. Compared with public law relying solely on administrative control, the easement model of natural resource protection adjusted by private law can produce better results, because the easement is generated voluntarily [74]. In addition, the advantages of the protective easement system are also reflected in broadening the scope of the subject of resource protection, reducing the cost of natural resource protection, balancing the interests of multiple subjects, etc. [49,76]. It can be seen from some legislation and practices in developed countries that although this system has played a significant role in the field of ecological protection, there are still some limitations in the system itself, such as the aforementioned lack of flexibility in the protection of easement, the difficulty in assessing the value of land, and the ambiguity of protection objectives [33,46,77]. At present, the land trust has become the main force of natural resource protection in many countries. Although the government is also directly involved in many conservation easement agreements, they do not intend to invest too much money [27,78]. This transfer of responsibility for natural resource protection further illustrates the decentralization of natural resource management and the weakening of government participation [35,76].

According to the existing literature, the advantages of the system of conservation easement and public law complement each other more than the defects of the system. Today, China has also begun to explore the decentralization of natural resource protection responsibilities. Compared with other countries, China still has many congenital deficiencies. As early as 1980, the United States Senate pointed out in its report that we should pay attention to the role of conservation easement in the protection of natural resources and subsequently promulgated the Conservation Easement Act, which established the basic content of the conservation easement system and gave practical policy preferences to important conservation easements [79]. Up to now, servitude has been mentioned in legal provisions and government regulations such as the property rights section of the Civil Code in China. However, there are no specific legal provisions on how to use conservation easements. Under the background of the long-term lack of a private law system, overcoming the existing defects, creating a natural protection easement model that suits the Chinese situation, and providing effective economic incentives for land users and private institutions to protect their land are the key issues related to the effect of ecological protection.

Therefore, scholars should attach great importance to the Chinese situation of the research object when studying the land trust issue in China; fully consider the policy background of China to accelerate the reform of the ecological civilization system and build a beautiful China, as well as the current situation of institutional arrangements for the coexistence of state ownership and collective ownership of land in nature reserves; conduct in-depth research on Chinese samples of natural reserve land trusts (such as Qianjiangyuan National Park pilot project and Wuyishan National Park pilot project) [80,81]; and summarize and refine the land trust protection model that conforms to China’s national conditions and can be replicated and popularized, so as to provide a valuable decision-making reference for governments at all levels.

## 5. Conclusions

Based on the 1476 English literature data in the WoS core database, this paper uses VOSviewer and CiteSpace software to analyze the literature visualization map of land-trust-related research. The analysis finds the following: (1) According to the publication trend since 1997, international nature reserve land trust research has progressed through three stages: beginning, development, and maturity. The number of related papers published generally shows an exponential growth trend, and the research shows the characteristics of multidisciplinary cross-integration. (2) In terms of research hotspots, land trusts and private land protection, the impact of and response to climate change, and the trust mechanism in collaborative governance are the three hot topics of international land trust research, with the first two hot topics being relatively closely related. (3) From the perspective of the research knowledge base, international land trust research has formed seven subject knowledge clusters with clear boundaries, among which the literature under the conservation easement cluster constitutes the beginning of a series of research on land trusts. This key literature has played a leading role in the evolution of the knowledge base of land trust research. (4) According to the existing literature, the advantages of the system of conservation easement and public law complement each other more than the defects of the system. Land trust conservation easement as private law can effectively make up for the deficiency of public law.

After decades of development, international land trust research has shown a trend of giving priority to empirical research and giving consideration to normative research. However, most of the existing research on land trusts in China is qualitative speculative research, and quantitative empirical research is relatively scarce, so it is difficult to provide a scientific and reasonable theoretical explanation for some problems faced in the development of land trusts. An important reason for this situation is that land trust conservation is still a new thing in China, and domestic scholars are still in the exploratory stage for quantitative empirical research on land trusts. At this stage, it is suggested that domestic scholars learn from the mature theoretical framework and empirical research methods of international land trust research, obtain the original data of China’s land trust protection projects through rigorous technical route design and objective and careful observation, and put forward theoretical assumptions and empirical tests on the difficult problems in land trusts, so as to effectively make up for the deficiencies of domestic quantitative empirical research on land trusts and consolidate the knowledge base of land trust protection research in China.

## Figures and Tables

**Table 1:** The keywords and co-occurrence calculation.

Cluster	Keywords (Occurrence Counts)
#1	land trust (66); conservation easement (51); land use (24); private land conservation (18); land conservation (16); attitudes (16)
#2	conservation (42); biodiversity (25); ecosystem services (25); protected area (21); climate change (16)
#3	trust (67); social capital (38); community land trust (33); sustainability (27); governance (27); collaboration (19); China (18); Kenya (17); land (16); indigenous (16); agriculture (15); property rights (15); participation (15)

**Table 2:** Specific information of clusters.

Cluster	Cluster Name	Size	Year	Silhouette
#0	conservation easement	47	2004	0.968
#1	land acquisition	39	2009	0.948
#2	knowledge exchange	39	2014	0.989
#3	wildlife interaction	38	2017	0.992
#4	water quality	37	2014	0.994
#5	land trusts	33	2012	0.98
#6	social capital	30	2015	0.982

**Table 3:** Land trust documents with the strongest citation bursts.

Publisher	Article	Year	Burst Value	Start	End
Massachusetts: Lincoln Institute of Land Policy	Reinventing Conservation Easements: A Critical Examination and Ideas for Reform	2005	3.51	2006	2008
Conservation Biology	Land Trusts and Conservation Easements: Who Is Conserving What for Whom?	2004	7.01	2007	2009
Natural Areas Journal	Conservation easements as a conservation strategy: Is there a sense to the spatial distribution of easements	2005	4.66	2007	2009
Conservation Biology	Conservation Easements: Biodiversity Protection and Private Use	2007	6.45	2008	2011
Massachusetts: The MIT Press	Buying Nature: The Limits of Land Acquisition as a Conservation Strategy, 1780–2004	2005	3.5	2008	2010

Note: The start and end years indicate the start and end years of a sudden increase in the citation frequency of a document.

## Data Availability

Not applicable.
